# Effect of TiO_2_ Nanoparticles on Tensile Strength of Dental Acrylic Resins

**DOI:** 10.5681/joddd.2014.036

**Published:** 2014-12-03

**Authors:** Saeed Shirkavand, Elnaz Moslehifard

**Affiliations:** ^1^Assistant Professor, Department of Prosthodontics, Faculty of Dentistry, Urmia University of Medical Sciences, Urmia, Iran; ^2^Dental and Periodontal Research Center, Tabriz University of Medical Sciences, Tabriz, Iran; ^3^Assistant Professor, Department of Prosthodontics, Faculty of Dentistry, Tabriz University of Medical Sciences, Tabriz, Iran

**Keywords:** Acrylic resins, materials testing, metal nanoparticles, tensile strength

## Abstract

***Background and aims.*** Adding further fillers to dental resins may enhance their physical characteristics. The aim of this study was to evaluate the tensile strength of heat-curing acrylic resin reinforced by TiO2nanoparticles added into the resin matrix.

***Materials and methods.*** Commercially available TiO_2_ nanoparticles were obtained and characterized using X-ray diffrac-tion (XRD) and scanning electron microscopy (SEM) to determine their crystalline structure, particle size and morphology. TiO_2_-acrylic resin nanocomposite was prepared by mixing 0.5, 1 and 2 (wt%) of surface modified TiO_2_ nanoparticles in an amalgamator providing three groups of samples. Before curing, the obtained paste was packed into steel molds. After cur-ing, the specimens were removed from the molds. The tensile strength test samples were prepared according to ISO 1567.

***Results.*** Two crystalline phases were found in TiO_2_ nanoparticles including: (i) anatase as the major one, and (ii) rutile. The average particle size calculated according to the Scherrer equation was 20.4 nm, showing a normal size distribution. According to SEM images, the nanocomposite with 1wt% TiO_2_ nanoparticles had a better distribution compared to other groups. In addition, the group by 1wt% TiO_2_ exhibited higher tensile strength with a significant difference compared to other groups. ANOVA showed significant differences between the contents of TiO_2_ particles in acrylic resin (F = 22.19; P < 0.001).

***Conclusion.*** A considerable increase in tensile strength was observed with titania NPs reinforcement agents in 1wt% by weight. Further increase of TiO_2_ nanoparticles decreased the tensile strength.

## Introduction

Heat-curing acrylic resins are frequently used in temporary prosthetic-base materials, provisional prosthesis, and orthodontic removable appliances such as retainers and functional appliances.^1-2^ Polymethylmethacrylate (PMMA) is generally used as a common component of acrylic materials due to its optical properties, biocompatibility and aesthetics.^3^ However, low mechanical properties against impact, bending, and fatigue are important issues to be addressed in order to improve acrylic polymers properties for removable acrylic appliances and dentures.^4^ Various methods have been used for improving mechanical properties such as chemical correction of polymeric structure by additives like polyethylene glycol dimetacrylate.^5^ The other useful method is to reinforce acrylic base composite by materials like fibers and particles.^6-8^

Manufacturers traditionally reinforce polymers with micrometer-size fillers to gain higher strength and stiffness, to improve solvent or fire resistance, or simply to reduce cost. However, these microfillers also impart several drawbacks such as brittleness and opacity. Nano-composites with at least one dimension less than 100 nm provide a new way to overcome the limitations of traditional composites.^9^ One can distinguish three categories of nano-composites, depending on the geometry of nanofillers including one dimensional (1D) nano-tube fillers such as carbon nano-tubes, two dimensional (2D) plate-like nanofillers such as clay, and nano-powders such as nano-alumina or nano-silica.^10-13^ These reinforcement agents have been added to the polymerizing matrix in order to improve the fatigue properties and fracture toughness of the PMMA. These include ﬁbers made of Kevlar, polyethylene, carbon, hydroxyapatite, bone mineral, high-strength PMMA ﬁbers and titanium as well as particles of glass, alumina andacrylonitrile-butadine-styrene.^13-19^

Among many nanocomposite constituents, TiO_2_ nanoparticles are increasingly used owing to their non-toxic, chemically inert, and low cost, high refractive index, antibacterial characteristic under a variety of spectrum, corrosion resistant and high hardness.^20^ Literature has also showed that nanoscale TiO_2_ reinforcement agents brings new optical, electrical, physiochemical properties attained at very low TiO_2_ content, which makes polymer-TiO_2_ nanocomposites a promising new class of materials.^20-22^ One can foresee that they will be commercially beneficial for widespread fields. Nevertheless, the study of TiO_2_-based nanocomposites is still in its infancy and much research remains to be carried out to explore improved synthesis techniques yielding the different nanocomposite structures and to fully understand the actual structure/properties relationships.

This study aims to investigate the effect of addition of TiO_2_ nanoparticles on the acrylic resin properties including mainly morphology, structure and tensile strength and modulus of toughness and elastic modulus.

## Materials and Methods

The heat-curing acrylic resins used in this study were PMMA (supplied by Ivoclar Vivadent). TiO_2_ nanoparticles with an average diameter less than 25 nm were purchased from the Anataz, TiO_2_, Nanosav, Tehran, Iran.

Surface treatment of TiO_2_ nanoparticles was carried out as follows: TiO_2_ nanoparticles were mixed with the solid acrylic resin polymer in an amalgamator to obtain three different composites with 0.5, 1 and 2wt% TiO_2_. To obtain a good distribution of TiO_2_ nanoparticles in the acrylic resin, the mixing procedure were continued for up to 20 min. Then, the mixed solid powder was manually blended with the resin monomer and then the mixture was then thoroughly blended. Before curing, the resulting paste was packed into steel molds using a vibrator to remove possible air bubbles. After curing, the specimens were removed from the molds.

The sample dimensions were selected according to standard ISO 1567 for comparing the tensile strength of samples with the control level.^23^ Sample size was determined according to a pilot study carried out on 4 samples. Using power-spread with α = 0.05 and power = 80, the results obtained from the pilot study indicate that 9 samples are required in each group. Four study groups, 9 samples were prepared for each test and totally, 36 samples to determine the mean number of specimens required for tensile strength tests.

The TiO_2_ nanoparticles were characterized using XRD (Philips X’pert, Cu Ka radiation) for crystalline structure and particle size. Also, scanning electron microscopy (SEM, VEGA/TESCAN) was used to study morphology of samples.

**Figure 1.**
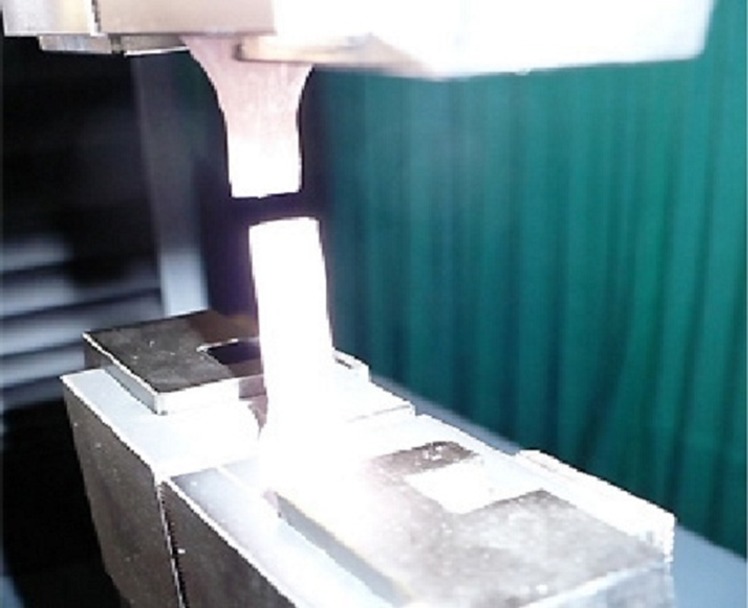
An image of a tested sample and tensile test set-up used in the experiments.

Each specimen was tested for tensile strength according to ISO 1567. Specimen plates were formed into a rectangle (60 × 12 × 4 mm) with a metal mold (n = 4). Rectangular specimens were then divided into 4 groups of 9 samples as follows: (1) acrylic resin as a control; (2) acrylic resin containing 0.5wt% TiO_2_; (3) acrylic resin containing 1wt% TiO_2_; and (4) acrylic resin containing 2wt% TiO_2_. Surfaces of all the specimens were polished using silicon carbide papers (mesh numbers of 500–2000). The tensile strength test was conducted with a universal testing machine (Zwick Z100, Germany) at a crosshead speed of 5mm/min under a load cell capacity of 10 kN to obtain stress-strain curves. Figure 1 shows the tested sample in test machine. Elastic modulus was determined according to the slope of the linear section of stress-strain curves. Modulus of toughness was calculated by measuring the entire area under the stress-strain curve from origin to rupture.

After testing, all results were analyzed by statistical methods. Mean, average, and mod in each group calculated and normal distribution curve was evaluated. Kolmogorov-Smirnov test was used to evaluate normal distribution. Statistical analysis of the obtained results for each test group was conducted using a one-way analysis of variance (ANOVA) followed by multiple comparison test (Scheffe’s test). Statistical significance was set at P < 0.05.

## Results

**Figure 2.**
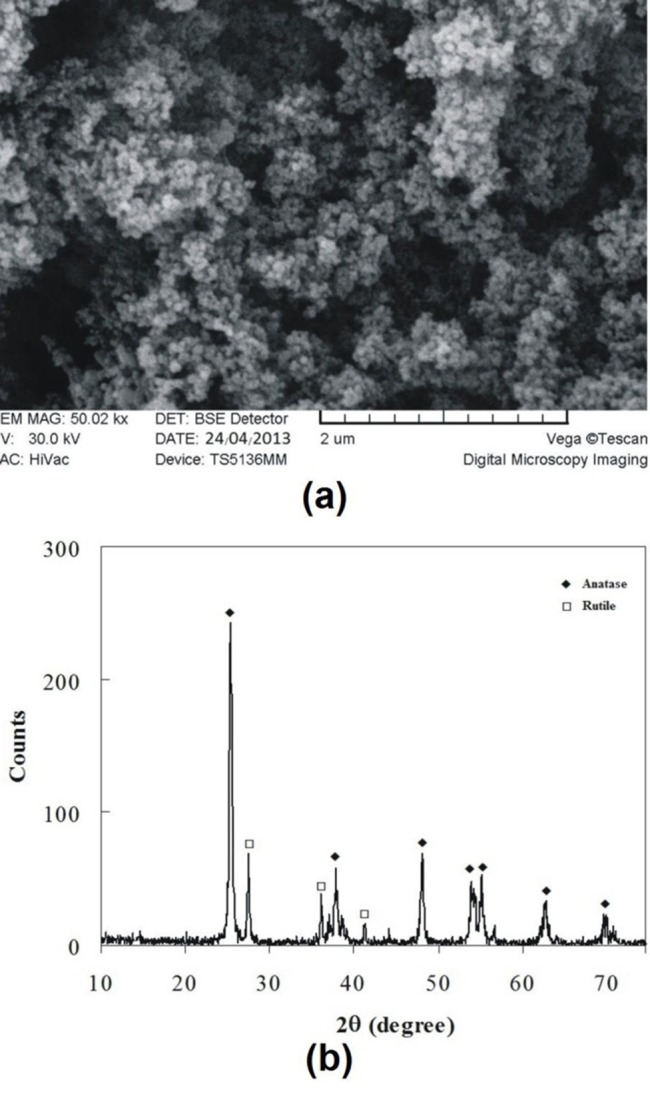
(a) SEM image, and (b) X-ray diffraction spectrum of TiO_2_Nanoparticles.

Figure 2a and 2b show SEM image and XRD spectrum obtained from the titanium oxide nanoparticles, respectively. Two crystalline polymorphs of titanium oxide with tetragonal crystal structures are present: (i) anatase and (ii) rutile. Anatase is found to be the major phase in the TiO_2_ sample, although a certain amount of rutile was also observed (Figures 2b). From X-ray diffractogram shown in  Figure 2b, the grain size of the TiO_2_ nanoparticles may be obtained by the Scherrer formula as:^24^ D = 0.9 λ/βcosθ where D, λ, β, and θ are grain size, wave length, peak width, and the angle, respectively. This equation shows reciprocal relation of broadening of peaks (β) in full width in middle height as the, grain size (D). Metal oxide particles range from this equation in various peaks, but for the highest peak the grain size is obtained 20.4 nm in diameter.

**Figure 3.**
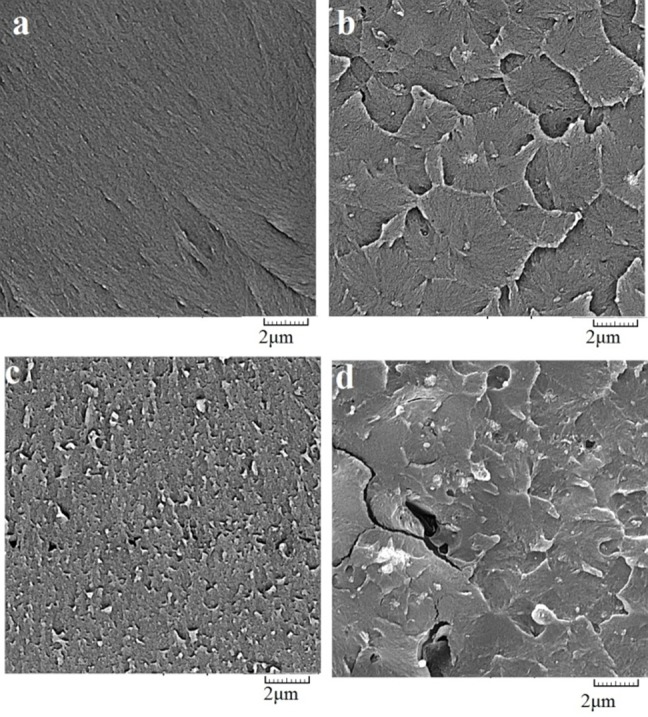
SEM images taken from cross-section of (a) control sample and nanocomposite containing TiO_2_ nanoparticles with (b) 0.5wt% (c) 1wt% , and (d) 2wt%.

The morphology of nanocomposite samples in cross section is shown in Figure 3. As seen in the SEM images, TiO_2_ at 1wt% has a better distribution compared to two other groups. Comparison between Figure 3cand3d revealed that an increase in TiO_2_ nanoparticle content from 1wt% to 2wt% caused tiny cracks inside the acrylic matrix. The cracks were observed in the as-prepared nanocomposite samples without application of any stressas seen in previous report.^25^

**Figure 4.**
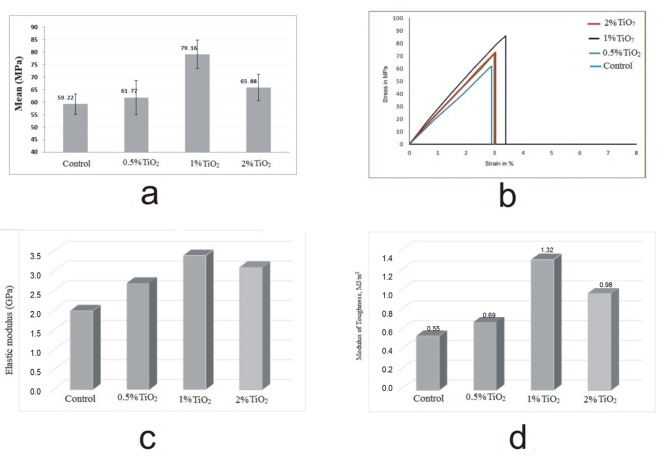
Mean values and standard deviations of tensile strengths calculated for all specimens (a). Stress-strain curve obtained for samples in tensile strength test (b). Elastic modulus of control and nanocomposite acrylic resins in mean values (c). Modulus of toughness of control and nanocomposite resins in mean values (d).

The mean and standard deviation of tensile strength measured for all groups along with ANOVA statistics are shown in Figure 4a and Table 1. According to the results of ANOVA followed by Scheffe’s test summarized in Table 2, there is a significant difference between the group of 1wt% TiO_2 _and other studied groups.

**Table 1:** Results measured for tensile strength of specimens with ANOVA statistics.

					One way ANOVA	
Sample	Numbers	Mean square ± standard deviation	Min	Max	F value	P value
acrylic resin	9	59.22 ± 4.02	52.00	64.00	22.916	0.001
acrylic resin with TiO_2_=0.5%	9	61.77 ± 6.81	53.00	72.00		
acrylic resin with TiO_2_= 1%	9	79.16 ± 5.68	72.00	87.00		
acrylic resin with TiO_2_=2%	9	65.88 ± 5.34	59.00	73.00		

In Figure 4b, typical stress-strain curve obtained for various tested samples of different groups are shown. The mean tensile strength was 59.22 ± 4.02 Mpa (range, 52-64 Mpa) in acrylic resin without additive, 61.77 ± 6.81Mpa (range, 53-72 Mpa) in the nanocomposite with 0.5wt% TiO_2_, 79.16 ± 5.68 Mpa (range, 72-87 Mpa) in the nanocomposite with 1wt% TiO_2_, and 65.88 ± 5.34 Mpa (range, 59-73 Mpa) in the nanocomposite with 2wt% TiO_2_. According to the results, reinforced acrylic resin with TiO_2_ nanoparticles exhibited an increased tensile strength up to 35wt% in maximum magnitude. ANOVA test showed significant differences between different contents of TiO_2_ nanoparticles (F = 22.19; P < 0.001). Multiple comparison test (Scheffe’s test) results for comparing tensile strength in various groups are listed in Table 2. These results show that acrylic resin having 1wt% TiO_2_ has the maximum tensile strength significantly compared to the two other groups. Also, the other groups did not show significant differences according the Scheffe’s test. Figures 4c and 4d show the elastic modulus and modulus of toughness achieved for the control and nanoparticles-reinforced acrylic resin in mean value, respectively. These results were extracted from the corresponding stress-strain curves.

**Table 2:** Multiple comparison test (Scheffe’s test) results to compare the tensile strength in various groups.

				95% confidence level.	
Group (I)	Group (J)	The mean difference (I-J)	P value	The lower limit	The upper limit
acrylic resin	TiO_2_ 0.5wt%	-2.56	0.81	-10.29	5.18
	TiO_2_ 1wt%	-19.94^*^	0.00	-27.68	-12.21
	TiO_2_ 2wt%	-6.67	0.11	-14.40	1.06
acrylic resin withTiO_2_=0.5wt%	acrylic resin	2.56	0.81	-5.18	10.29
	TiO_2_ 1wt%	-17.38^*^	0.00	-25.12	-9.66
	TiO_2_ 2wt%	-4.11	0.49	-11.84	3.62
acrylic resin with TiO_2_=1wt%	acrylic resin	19.94^*^	0.00	12.21	27.68
	TiO_2_ 0.5wt%	17.38^*^	0.00	9.66	25.12
	TiO_2_ 2wt%	13.27^*^	0.00	5.55	21.01
acrylic resin with TiO2=2wt%	acrylic resin	6.67	0.11	-1.06	14.40
	TiO_2_ 0.5wt%	4.11	0.49	-3.62	11.84
	TiO_2_ 1wt%	-13.27^*^	0.00	-21.01	-5.55

## Discussion

Important physical properties of acrylic resins are shown to be influenced by the addition of TiO_2_ nanoparticles as the reinforcement agents. In recent years, metal oxide nanoparticles have been largely investigated for their activity as antimicrobial additives. In particular, TiO_2_ is now considered a low-cost, clean photocatalyst with chemical stability and non-toxicity, and has been used for a wide variety of environmental applications, including water treatment and air purification.^26,27^ Acosta-Torre et al^28^ reported that the introduction of nano-sized metal oxide materials for preparing acrylic resins allows the production of polymer with both color and surface modifications. According to the latter study, physical properties of nanopigmented and standard PMMA showed a lower porosity for TiO_2_ containing PMMA. This finding suggests that the metal oxide nanoparticles are suitable additives for the improvement of PMMA formulations, since high porosities have been considered a critical drawback for PMMA in prosthodontics applications.^28^ The authors of the latter study suggested that nanotechnology-assisted design allows a product with well-controlled morphology. The study of mechanical properties also showed that PMMA-containing nanoparticles behave as is specified by the International Standards for Denture Prosthetics.^28,29^ This study was conducted to test the effects of TiO_2_ on tensile strength of final acrylic product.

Based on our obtained tensile strength values, incorporation of nano-sized TiO_2_ at various contents of 0.5, 1 and 2wt% affected the tensile strength of the polymerized material, where the tensile strength increased with increasing the concentration of TiO_2_ nanoparticles up to 1wt% and then decreased by an additional amount of TiO_2_nanoparticles introduced into the nanocomposite resin. These results are consistent with previous studies.^26,30^ The additional TiO_2_ nanoparticles act as impurities and the tensile strength decreases as a result of the extra additive. Improper dispersion of TiO_2_ in acrylic PMMA matrix unfavorably affects the reaction of monomers, leading to increased levels of unreacted monomer, which acts as plasticizer.^26,31^ The content of nanoparticle additives is of critical importance. When TiO_2_ nanoparticles exceeded a particular percentage, i.e. 1wt% in this study, the opposite trend of decreased tensile strength was observed.

The increase in TiO_2_ content causes these particles to agglomerate. The agglomerated compounds can act as stress concentrating centers in the matrix and adversely affect the mechanical properties of the polymerized material.^30,32^ Preventing agglomeration has been a main challenge in nanocomposite production. Scanning electron microscopy was carried out for all samples in order to study this effect (Figure 3). The agglomeration of TiO_2_ probably gives rise to some micro-pores and micro-crack as structural defects. These defects were not present in low percentages of TiO_2_ nanoparticles. Micro-cracks and micro-pores are caused by stress concentration sites and loss of mechanical properties. Formation of the cracks could be due to higher stress levels in the acrylic resin induced by more TiO_2_ particles reinforced in the bulk polymer. The cracks are not a result of external stress but due to TiO_2_/polymer interface energy.^25^

The modulus of toughness measured for the nanocomposite acrylic resin increased by about 1.4 times over the control group by the addition of TiO_2_ nanoparticles (Figure 4d). Elastic modulus of reinforced resin with 1wt% TiO_2_ nanoparticles was at least 70wt% higher than that of the control acrylic resin sample (Figure 4c). The increase in the elastic modulus was the highest for 1wt% TiO_2_ while it was the lowest when a high value was added to the polymerizing matrix, due to the effect of defects discussed earlier. The increased porosity in 2wt% TiO_2_ nanocomposite impairs its tensile strength (elastic modulus and strength). There is a trend towards a decrease in the modulus of toughness and elastic modulus with TiO_2_ nanoparticles content at higher concentrations. This finding explains the dysfunctional effect of TiO_2_ in higher amounts and also the importance of content control of TiO_2_ nanoparticles.

## Conclusion

A significant increase in tensile strength values were observed in TiO_2_ nanoparticles-acrylic resin containing 1wt% TiO_2_. Further increase in TiO_2_ nanoparticles content impose an inverse effect by decreasing the tensile strength due to the agglomeration of particles acting as defects and stress concentrated points.

## Aknowledgement

This article was written based on a dataset from an MSc thesis entitled “Comparison of compressive strength; tensile strength and impact strength of reinforced acrylic resin with TiO_2_ nanoparticles and conventional acrylic resin” registered at Tabriz University of Medical Sciences Faculty of Dentistry (reference number 160/T). The thesis was supported by the Vice Chancellor for Research at Tabriz University of Medical Sciences.
